# Identification of diagnostic biomarkers correlate with immune infiltration in extra-pulmonary tuberculosis by integrating bioinformatics and machine learning

**DOI:** 10.3389/fmicb.2024.1349374

**Published:** 2024-02-07

**Authors:** Yanan Wang, Faxiang Jin, Weifang Mao, Yefu Yu, Wenfang Xu

**Affiliations:** Department of Clinical Laboratory, Affiliated Hospital of Shaoxing University, Shaoxing, Zhejiang, China

**Keywords:** extra-pulmonary tuberculosis, WGCNA, LASSO, SVM-RFE, biomarker

## Abstract

The diagnosis of tuberculosis depends on detecting Mycobacterium tuberculosis (Mtb). Unfortunately, recognizing patients with extrapulmonary tuberculosis (EPTB) remains challenging due to the insidious clinical presentation and poor performance of diagnostic tests. To identify biomarkers for EPTB, the GSE83456 dataset was screened for differentially expressed genes (DEGs), followed by a gene enrichment analysis. One hundred and ten DEGs were obtained, mainly enriched in inflammation and immune -related pathways. Weighted gene co-expression network analysis (WGCNA) was used to identify 10 co-expression modules. The turquoise module, correlating the most highly with EPTB, contained 96 DEGs. Further screening with the least absolute shrinkage and selection operator (LASSO) and support vector machine recursive feature elimination (SVM-RFE) narrowed down the 96 DEGs to five central genes. All five key genes were validated in the GSE144127 dataset. CARD17 and GBP5 had high diagnostic capacity, with AUC values were 0.763 (95% CI: 0.717–0.805) and 0.833 (95% CI: 0.793–0.869) respectively. Using single sample gene enrichment analysis (ssGSEA), we evaluated the infiltration of 28 immune cells in EPTB and explored their relationships with key genes. The results showed 17 immune cell subtypes with significant infiltrations in EPTB. CARD17, GBP5, HOOK1, LOC730167, and HIST1H4C were significantly associated with 16, 14, 12, 6, and 4 immune cell subtypes, respectively. The RT-qPCR results confirmed that the expression levels of GBP5 and CARD17 were higher in EPTB compared to control. In conclusion, CARD17 and GBP5 have high diagnostic efficiency for EPTB and are closely related to immune cell infiltration.

## 1 Introduction

Tuberculosis (TB) is caused by infection with Mtb. According to the World Health Organization's (WHO) Global Tuberculosis Report 2022, an estimated 10.6 million people developed TB in 2021 compared with 10.1 million in 2020. The mortality rate in 2021 is also on an increasing trend compared to 2020 (Bagcchi, 2023). EPTB refers to Mtb colonizing and growing in organs other than the lungs and causing tuberculosis-like pathological changes, such as spinal tuberculosis and renal tuberculosis. Pulmonary tuberculosis (PTB) and extra-pulmonary tuberculosis (EPTB) can co-exist (Ohene et al., 2019), and the rate of drug resistance is even higher in EPTB (Boonsarngsuk et al., 2018). Unfortunately, current research on biomarkers of TB is focused on PTB, with less attention paid to EPTB (Sanches et al., 2015).

Because of the atypical clinical features and symptoms, the diagnosis of EPTB relies on pathological examination by biopsy or puncture extraction. Whereas tissue specimens are difficult to digest and have a low bacterial load, traditional detection methods are not sensitive enough (Purohit and Mustafa, 2015). Several studies have suggested that some cytokines, such as tumor necrosis factor-alpha (TNF-α) (Ranaivomanana et al., 2018), interferon-gamma (IFN-γ) (Ranaivomanana et al., 2018; Antonangelo et al., 2019; Wani et al., 2021), IL-27 (Antonangelo et al., 2019) and IL-10 (Ranaivomanana et al., 2018; Wani et al., 2021) could be used as diagnostic biomarkers for EPTB. Inflammatory biomarkers are also altered before and after EPTB treatment (Vinhaes et al., 2019). Immunity plays a vital role in whether or not a host becomes infected with Mtb. Immunocompromised Individuals with a history of close contact with TB are at risk of developing TB (Boom et al., 2021). However, few studies have focused on biomarkers associated with immune infiltration of EPTB (Bhattacharya et al., 2017; Du Bruyn et al., 2022). Developing new serological markers of EPTB is of great importance for the global control of TB.

**Figure 1:**
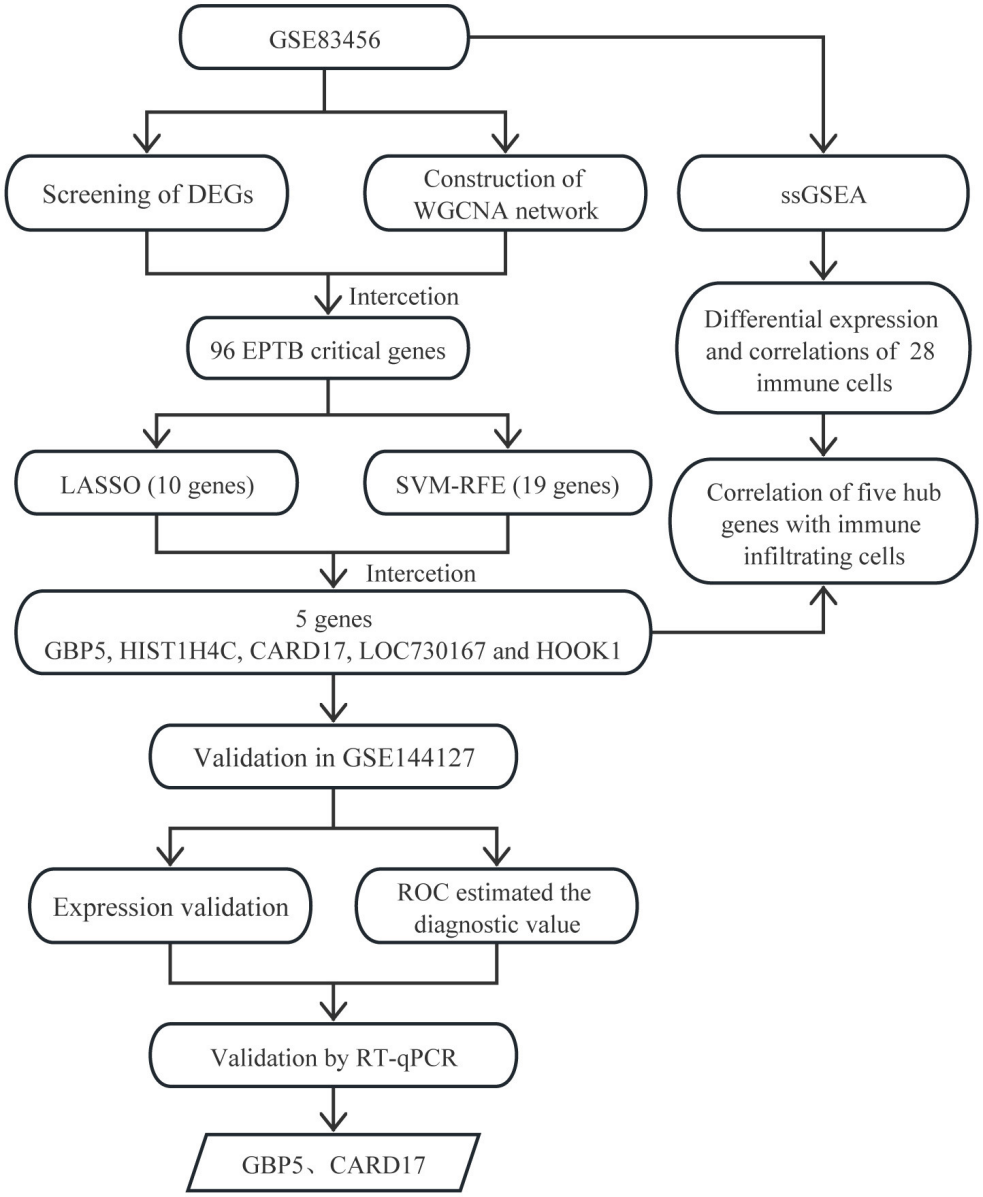
Flowchart of the identification of biomarkers correlate with immune infiltration in EPTB.

Recently, with the evolution and application of gene chips, bioinformatics techniques can help us quickly find the central gene clusters of EPTB. WGCNA is a bioinformatics analysis method that integrates all data from each sample in the dataset, including gene expression and clinical information data. WGCNA is mainly used to construct unordered networks and conduct association analysis by normalized data from each sample. It is now widely used to identify and screen for markers of complex diseases (Zhang and Horvath, 2005). LASSO is a regression method that elucidates the degree of association between two related variables (Cheung-Lee and Link, 2019). SVM is a supervised learning algorithm, primarily used in classification, regression and outlier detection. The RFE algorithm selected the optimal genes from the metadata queue to avoid over-fitting (Huang et al., 2018). Further LASSO and SVM-RFE analysis of the WGCNA gene can improve the accuracy of screening for signature-related genes (Duan et al., 2016). Based on the immune response to EPTB, we used ssGSEA to analyze the infiltration of 28 immune cells. WGCNA combined with machine learning for biomarker screening has been widely used in other diseases (Xin et al., 2023), but not in EPTB. This study provides new ideas for further evaluation of EPTB biomarkers (Figure 1).

## 2 Materials and methods

### 2.1 Microarray data

We searched the GEO datasets with key word “extra-pulmonary tuberculosis” or “extra-pulmonary TB,” restricted “entry type” to “series”; selected “Homo sapiens” for “organization”; selected “study type” for “Expression profiling by array.” For the subsequent analyses, datasets were selected based on the inclusion criteria of blood as the sample type and containing more than 20 samples. GSE83456 (Blankley et al., 2016) was a screening dataset, including expression data from 47 EPTB patients and 61 controls. GSE144127 (Hoang et al., 2021) was used as a validation dataset, including 163 EPTB patients and 325 controls. Matrix data (GSE83456 and GSE144127) and microarray annotation platform files were downloaded from the Gene Expression Omnibus (GEO) database.

### 2.2 Screening of differentially expressed genes

Use the “limma” package (Ritchie et al., 2015) in R4.1.3 for differential expression analysis. The screening criteria were |logFC| ≥1.0 and adj. *p* < 0.05. The volcano and heat maps were created using the “ggplot2” and “pheatmap” packages respectively.

### 2.3 Gene function enrichment analysis

GO and KEGG analysis to explore the functionalities and pathways involved in DEGs. The analysis was carried out using the “clusterProfiler” and “enrichplot” package in R4.1.3. The GO analysis is visualized through the “ComplexHeatmap” (Gu et al., 2016). Enrichment is statistically significant if *q*-value < 0.05.

### 2.4 Weighted co-expression network construction and feature module selection

The correlation matrix in the model was constructed using the 'WGCNA' analysis package in R4.1.3 to calculate the neighborhood relationships for all genes and to determine the soft threshold size (Horvath and Dong, 2008; Mason et al., 2009). Based on the soft threshold, the disordered neighborhoods between genes are truncated and eventually converted into a topological overlap matrix (TOM) to gauge the network connectivity between genes (Yip and Horvath, 2007; Botía et al., 2017). Hierarchical clustering was carried out according to the degree of variation in TOM so that genes with a similar degree of difference in gene expression were included in the same gene module. The correlation coefficient between the modules and the samples was calculated by Pearson correlation analysis to identify the largest contribution module of WGCNA. The maximum module for the number of links is acquired by accumulating absolute values.

### 2.5 Machine learning screening for hub genes

We employed LASSO and SVM-RFE to further identify the hub genes of EPTB. The LASSO analysis was carried out using the “glmnet” package (Tibshirani, 1996; Engebretsen and Bohlin, 2019). Ten-fold cross-validation method was applied on LASSO regression analysis. The reaction type was set to binomial and α was set to 1. The smallest regularization parameter, lambda (λ), was selected by 10-fold cross-validation. The value of λ was substituted into the LASSO regression model, and the sum of the absolute values of all regression coefficients was less than or equal to λ. The regression coefficients of most genes were compressed to 0. Genes without 0 regression coefficients were considered to be key genes highly associated with EPTB.

SVM-RFE is a backward search algorithm (Sanz et al., 2018). It ranks each feature with a score by training the sample with the model. After removing the features with the minimum feature score, the model is trained again with the remaining features for the next iteration, and finally the desired number of features is selected. SVM-RFE reduces the dimensionality of the space by eliminating unnecessary features. The SVM-RFE analysis was done by using the “e1071” (https://CRAN.R-project.org/package=e1071), “kernlab” (https://CRAN.R-project.org/package=kernlab) and “caret” packages (Kuhn, 2008). Internal cross-validation of the model is performed by SVM-RFE, using the method “svm Radial.”

### 2.6 Validation of hub gene expression and diagnostic value

The intersecting genes of LASSO and SVM-RFE were further validated in the GSE144127 dataset to confirm the expression and diagnostic value. The differential expression of hub genes in EPTB and controls was demonstrated using box plots. The receiver operating characteristic curve (ROC) estimated the diagnostic value using the “pROC” package (Robin et al., 2011).

### 2.7 Immune cell infiltration

The ssGSEA algorithm was used to determine the extent of immune cell infiltration in the GSE83456 dataset (Bindea et al., 2013). The violin plot of the 28 immune infiltrating cells was visualized to show the differential expression. Spearman's correlation between key genes and 28 immune infiltrating cells was calculated and then visualized using the “ggplot2” package.

### 2.8 Reverse transcription quantitative polymerase chain reaction (RT-qPCR)

Whole blood samples were collected from 10 HC and 10 EPTB patients. The diagnosis of EPTB was conducted by two respiratory specialists. All participants participated in the study with informed consent. The study was approved by the Medical Ethics Committee of the Affiliated Hospital of Shaoxing University.

Total RNA was extracted from whole blood using the QIAamp^®^ RNA blood Mini kit (Qiagen, Germany), and the obtained RNA was reverse transcribed to cDNA using the HiFiScript cDNA Synthesis Kit (Kangweishiji Biotech, China). Finally, qPCR was performed using the NovoStart^®^ SYBR qPCR SuperMix Plus (novoprotein, China). The primers were designed and synthesized by Guangzhou RiboBio Co., LTD. The sequences for the primers targeting the genes are provided in Table 1. As a housekeeping gene, β-actin served to normalize the expression levels. The relative mRNA levels of GBP5 and CARD17 were calculated by the 2^−ΔΔCT^ method (Livak and Schmittgen, 2001).

**Table 1:** The primer sequences used for RT-qPCR.

**Primer**	**Sequence**
GBP5-F	GCTTGCCCAACTTGAAACAC
GBP5-R	CATTGACCATGATGCCACCT
CARD17-F	AGCAGCCAGATGATAAAGCACA
CARD17-R	TTCTTCCGGCCCTCAGACA
β-actin-F	TCAAGATCATTGCTCCTCCTGAG
β-actin-R	ACATCTGCTGGAAGGTGGACA

### 2.9 Statistics

R is a programming language for statistics and graphics. All data analysis, statistics and graphs involved in this paper are done by R 4.1.3. The significant difference between the two groups was determined by *t*-test. The WGCNA's key modules were identified using Pearson 's correlation coefficient. Spearman correlation analysis was conducted on hub genes and infiltrating immune cells. *p* < 0.05 was considered statistically significant.

## 3 Results

### 3.1 Differentially expressed genes in EPTB

**Figure 2:**
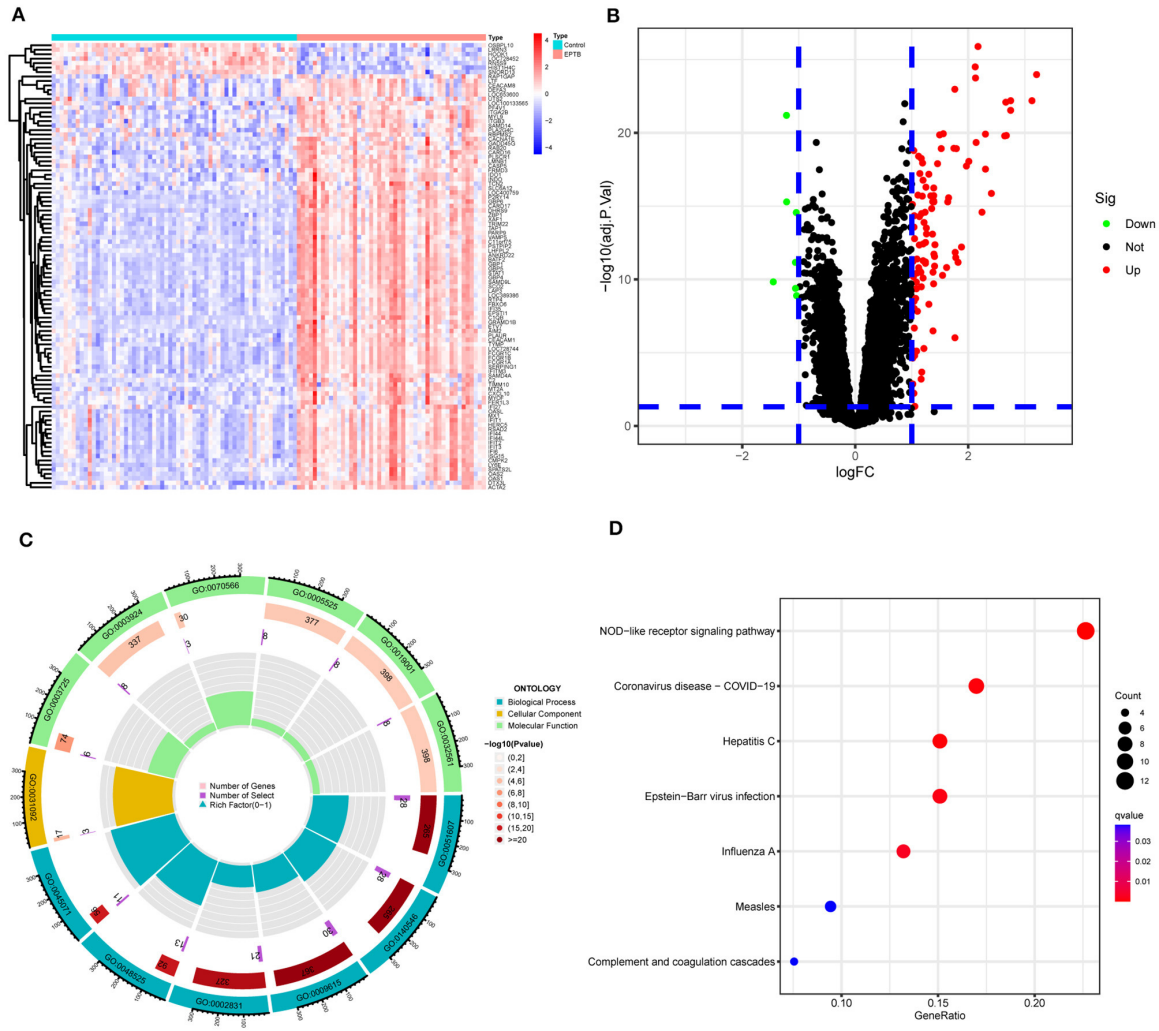
Screening and enrichment analysis of differentially expressed genes between EPTB and controls. **(A)** Heat map showing changes in the expression of DEGs. Blue indicates low expression and red indicates high expression. **(B)** Volcano plot showing DEGs between EPTB and control samples. Green indicates low expression and red indicates high expression. **(C)** GO analysis and **(D)** KEGG analysis.

The matrix data from the GSE83456 dataset and the GPL10558 platform annotation file were downloaded. After gene annotation and data pre-processing, the expression profiles of 31,300 genes were obtained. One hundred and ten DEGs were identified in EPTB compared to controls and consisted of 103 up-regulated genes and seven down-regulated genes. The heat map was constructed by performing a hierarchical cluster analysis based on DEGs screening (Figure 2A). The volcano plot in Figure 2B illustrated the distribution of DEGs.

### 3.2 Gene enrichment analysis

GO and KEGG analyses were performed on 110 DEGs to screen for significantly enriched functions and pathways at *q*-value < 0.05. GO analysis (Table 2 and Figure 2C) revealed that the biological processes involved in EPTB were mainly defense response to virus, defense response to symbiont and regulation of response to biotic stimulus. The main cellular component enriched by DEGs is the platelet alpha granule membrane. The molecular function is mainly related to double-stranded RNA binding, GTPase activity and adenylyl transferase activity. KEGG analysis (Table 3 and Figure 2D) showed that the relevant pathways were mainly involved in the NOD-like receptor signaling pathway and various virus-related pathways such as Hepatitis C, Influenza A, Coronavirus disease-COVID-19 and Epstein-Barr virus infection.

**Table 2:** GO analysis of differentially expressed genes in EPTB.

**ID**	**Description**	***p*-value**	***q*-value**
GO:0051607	Defense response to virus	0.000	0.000
GO:0140546	Defense response to symbiont	0.000	0.000
GO:0009615	Response to virus	0.000	0.000
GO:0002831	Regulation of response to biotic stimulus	0.000	0.000
GO:0048525	Negative regulation of viral process	0.000	0.000
GO:0045071	Negative regulation of viral genome replication	0.000	0.000
GO:0031092	Platelet alpha granule membrane	0.000	0.011
GO:0003725	Double-stranded RNA binding	0.000	0.000
GO:0003924	GTPase activity	0.000	0.036
GO:0070566	Adenylyltransferase activity	0.000	0.036
GO:0005525	GTP binding	0.001	0.036
GO:0019001	Guanyl nucleotide binding	0.001	0.036
GO:0032561	Guanyl ribonucleotide binding	0.001	0.036

**Table 3:** KEGG analysis of differentially expressed genes in EPTB.

**ID**	**Description**	***p*-value**	***q*-value**
hsa04621	NOD-like receptor signaling pathway	0.000	0.000
hsa05160	Hepatitis C	0.000	0.000
hsa05171	Coronavirus disease—COVID-19	0.000	0.001
hsa05169	Epstein-Barr virus infection	0.000	0.001
hsa05164	Influenza A	0.000	0.003
hsa05162	Measles	0.002	0.038
hsa04610	Complement and coagulation cascades	0.002	0.038

### 3.3 Weighted gene co-expression network analysis

**Figure 3:**
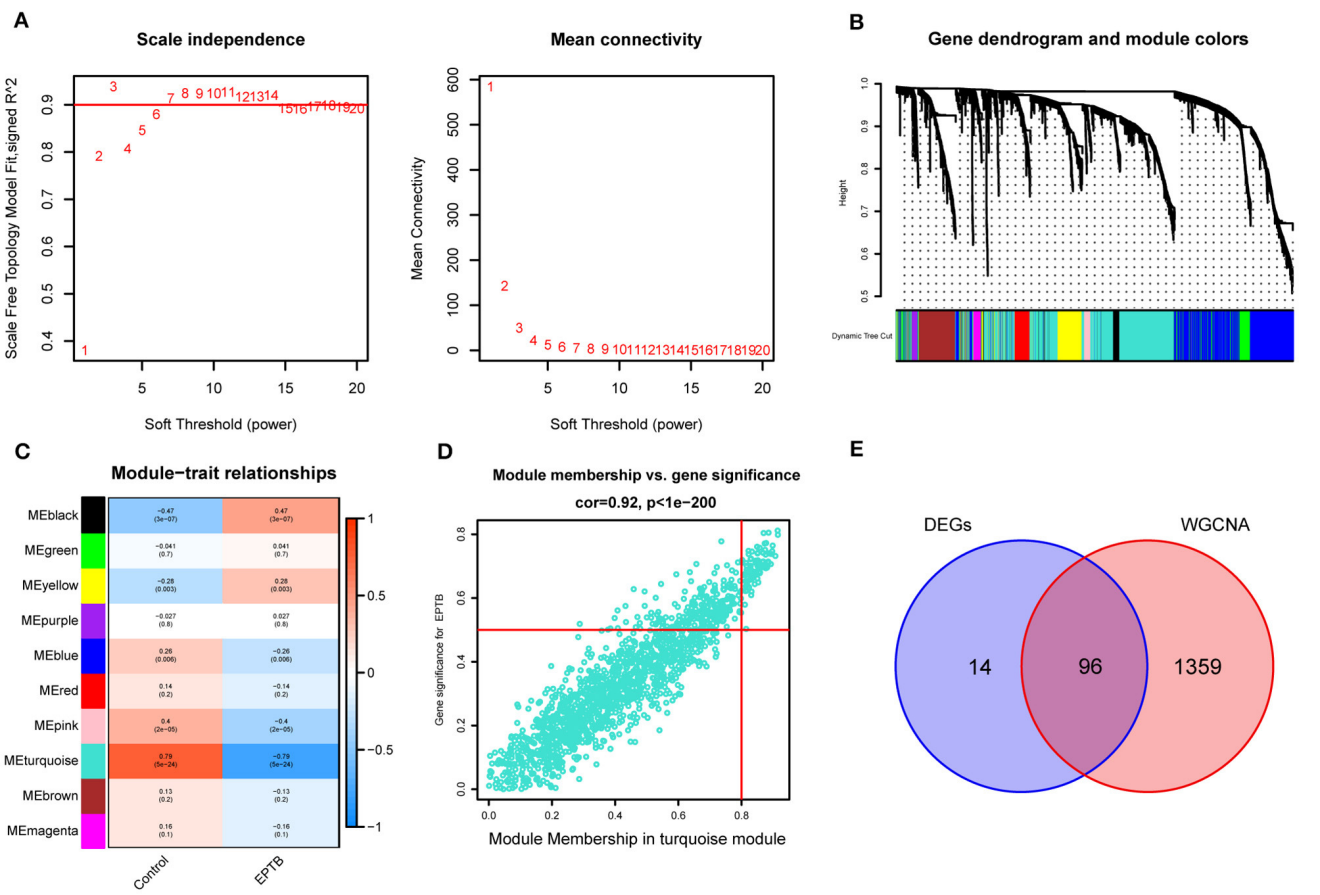
Construction of weighted gene co-expression network and module selection. **(A)** Scale-free network distribution plot, where the optimal power-law exponent (*β* = 3) is determined for subsequent network analysis. **(B)** Cluster tree diagram of genes. Each branch in the diagram represents a gene, and each color below represents a co-expression module. **(C)** Heat map of module-trait relationships. Turquoise module is the most strongly correlated with EPTB. **(D)** Scatter plot of correlations between genes and gene importance in the turquoise module. **(E)** Intersecting genes of differentially expressed genes and turquoise module genes.

The GSE83456 dataset was pre-processed to remove duplicate values and add missing values. Genes with expression fluctuations of < 0.5 were removed to reduce noise. According to the scale-free network distribution fit, β = 3 was automatically selected as the best soft threshold for this dataset using powerEstimate (Figure 3A). The adjacency matrix and topological overlap TOM between genes were calculated. A hierarchical clustering tree between genes was constructed based on the TOM. The modules with high similarity of MEs were merged using the dynamic shearing tree method to cluster the genes into 10 modules (Figure 3B). The 10 modules consist of the black module (122 genes), blue module (1,192 genes), brown module (382 genes), green module (298 genes), magenta module (79 genes), pink module (89 genes), purple module (57 genes), red module (168 genes), turquoise module (1,455 genes), and yellow module (348 genes). The genes that could not be clustered into any of the modules were grouped into Gray modules and removed in subsequent analyses.

According to Figure 3C, the Pearson correlation coefficient between the turquoise module and EPTB was 0.79 (*p* = 5e-24). With turquoise as the key module, GS and MM analysis was performed. The genes within the module were significantly linearly correlated with EPTB. The correlation coefficient was 0.92 (*p* < 1e-200), and the results are shown in Figure 3D. The co-expressed genes in the turquoise module were intersected with the DEGs to narrow down further, resulting in 96 EPTB critical pathogenic genes (Figure 3E).

### 3.4 Machine learning to identify EPTB key genes

**Figure 4:**
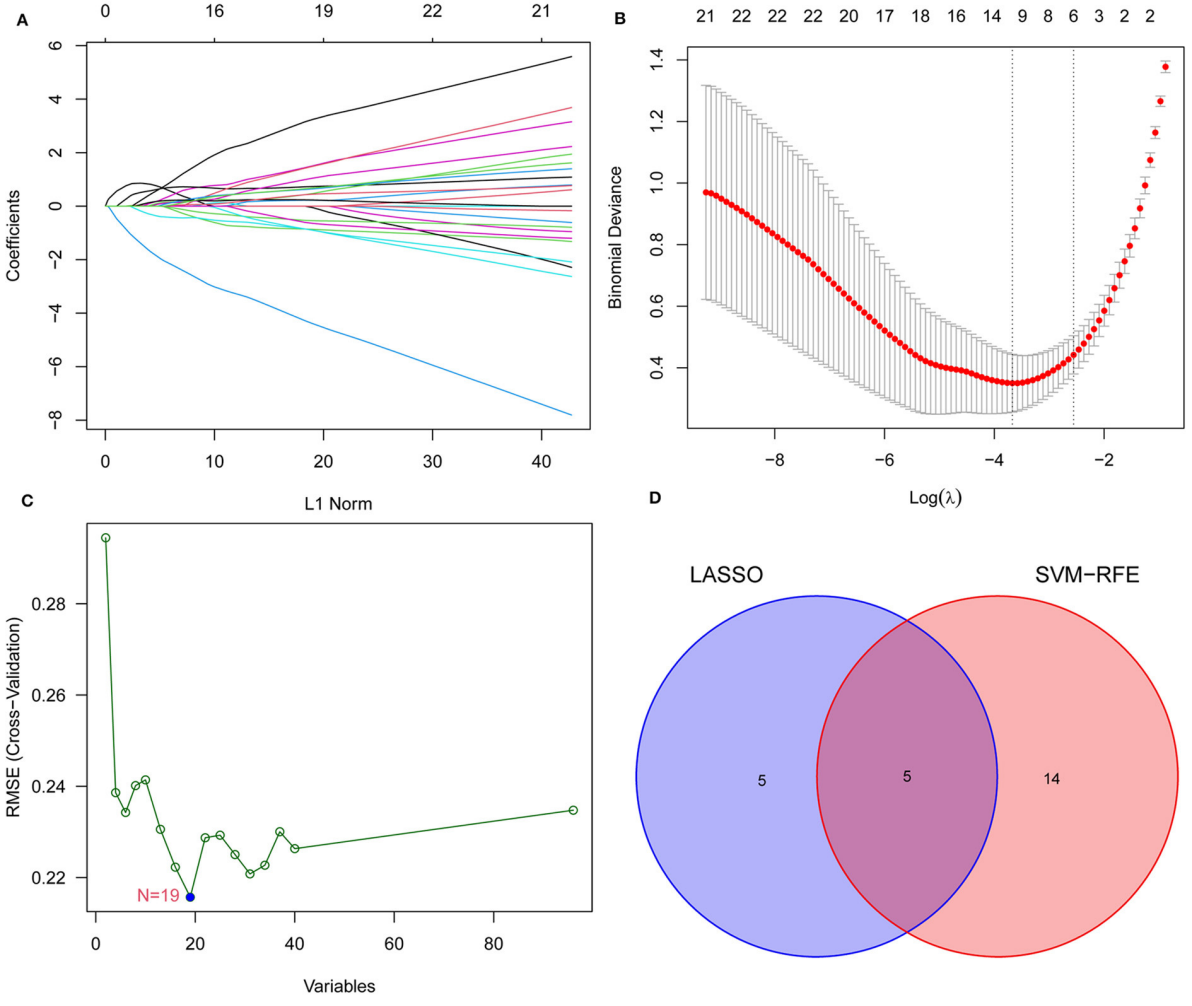
Narrowing down candidate biomarkers for EPTB by LASSO analysis and SVM-REF. **(A)** and **(B)** LASSO analysis. **(C)** SVM-REF analysis to identify signature genes. **(D)** Venn diagram of intersecting genes by LASSO analysis and SVM-REF.

We screened for EPTB biomarkers with two different machine learning algorithms. By using the LASSO regression, 10 variables were identified among the 96 overlapping genes (Figures 4A, B). The SVM-RFE identified 19 signature genes out of 96 intersecting genes (Figure 4C). Ultimately, five overlapping DEGs (GBP5, HIST1H4C, CARD17, LOC730167, and HOOK1) between the two algorithms were selected for the following analysis (Figure 4D).

### 3.5 Expression validation of characteristic biomarkers

**Figure 5:**
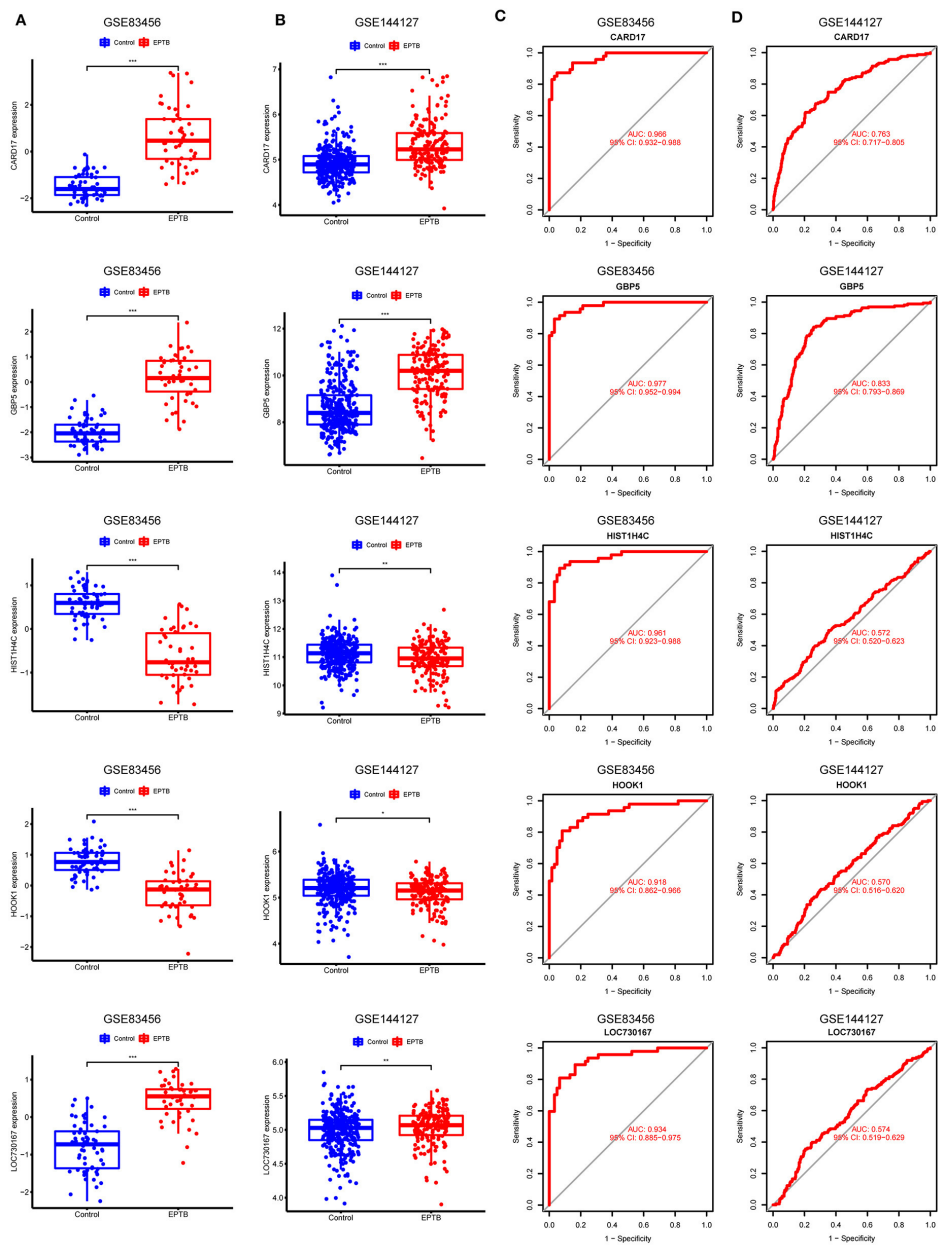
Validation and receiver operating characteristic curve of diagnostic biomarkers. **(A)** Expression of 5 hub genes in GSE83456. **(B)** Expression of 5 hub genes in the validation set GSE144127. **(C)** ROC curves for the 5 hub genes in GSE83456. **(D)** ROC curves for 5 hub genes in the validation set GSE144127.

CARD17, GBP5, and LOC730167 were over-expressed, while HIST1H4C and HOOK1 were under-expressed in the GSE83456 dataset, with all *p*-values < 0.001 (Figure 5A). Gene expression levels were validated by using 325 controls and 163 EPTB samples from the GSE144127 dataset. Consistent with the results of the GSE83456 dataset, the results showed high expression of CARD17 (*p* < 0.001), GBP5 (*p* < 0.001), and LOC730167 (*p* < 0.01), and low expression of HIST1H4C (*p* < 0.01) and HOOK1 (*p* < 0.05) in EPTB (Figure 5B). Although the differential expression of LOC730167, HIST1H4C, and HOOK1 in the GSE144127 dataset is still statistically significant, the extent of the difference is weaker compared to their expression in the GSE83456 dataset.

### 3.6 Diagnostic validity of characteristic biomarkers

The model performance was evaluated with well-known metrics area under the ROC curve (AUC). As shown in Table 4 and Figure 5C, the five biomarkers showed good diagnostic value in differentiating between EPTB and control samples (all the AUC > 0.9). The AUC was 0.977 (95% CI: 0.952–0.994) for GBP5, 0.966 (95% CI: 0.932–0.988) for CARD17, 0.961 (95% CI: 0.923–0.988) for HIST1H4C, 0.934 (95% CI: 0.885–0.975) for LOC730167 and 0.918 (95% CI: 0.862–0.966) for HOOK1. It was further validated in the GSE144127 dataset to confirm its diagnostic value, and the results are shown in Figure 5D. The AUC for GBP5 and CARD17was 0.833 (95% CI: 0.793–0.869) and 0.763 (95% CI: 0.717–0.805) respectively. Unfortunately, the diagnostic efficiency of HIST1H4C, LOC730167, and HOOK1 was poor. The AUC values for all three were < 0.6. Thus, we believe that GBP5 and CARD17 promise diagnostic markers for EPTB.

**Table 4:** The area under the ROC curve of hub genes.

**Gene**	**AUC (95%CI)**	
	**GSE83456**	**GSE144127**
GBP5	0.977 (0.952–0.994)	0.833 (0.793–0.869)
HIST1H4C	0.961 (0.923–0.988)	0.572 (0.520–0.623)
CARD17	0.966 (0.932–0.988)	0.763 (0.717–0.805)
LOC730167	0.934 (0.885–0.975)	0.574 (0.519–0.629)
HOOK1	0.918 (0.862–0.966)	0.570 (0.516–0.620)

### 3.7 Immune cell infiltration and its correlation with the hub gene

**Figure 6:**
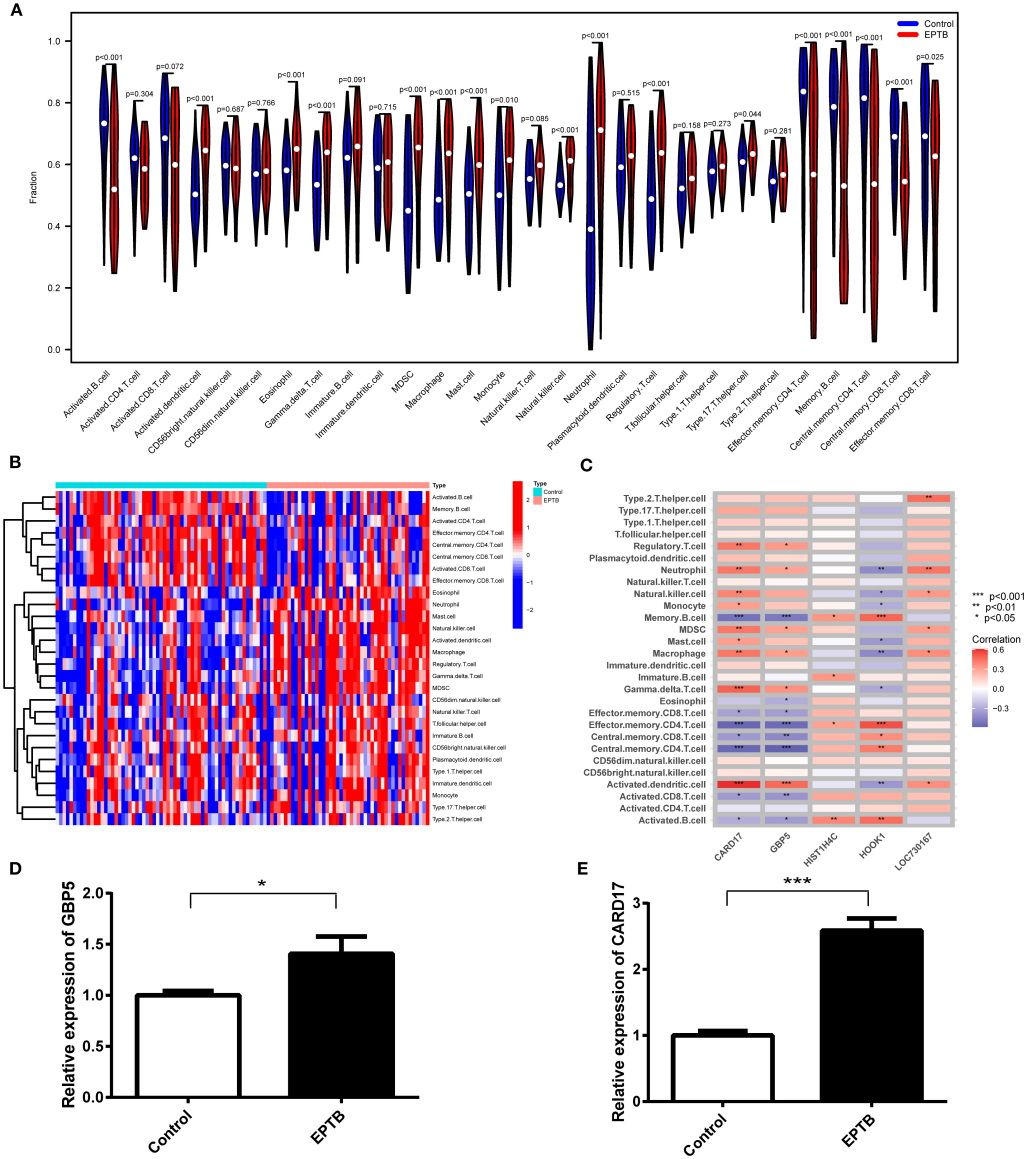
Analysis of immune infiltration associated with EPTB and RT-qPCR validation in clinical samples. **(A)** Violin plot of the 28 immune cells' distribution. **(B)** Heatmap of 28 immune cell correlations. **(C)** Correlation of five hub genes with immune infiltrating cells in EPTB. **(D)** Relative expression of GBP5 in EPTB compared to control. **(E)** Relative expression of CARD17 in EPTB compared to control (*p < 0.05, **p < 0.01, ***p < 0.001).

With the help of the ssGSEA algorithm, we assessed the differences between EPTB and healthy controls in regards to immune cell infiltration. The infiltration of 17 immune cell subtypes, including Memory B cells (*p* = 6.83e-6), Mast cells (*p* = 2.13e-5) and Regulatory T cells (*p* = 2.13e-5), was significantly different compared to healthy controls. It indicated an essential role of immune cells in the progression of EPTB (Figure 6A). The distribution of the 28 immune cells in the GSE83456 sample was shown in Figure 6B, and correlation analysis with the hub gene was shown in Figure 6C. CARD17, GBP5, and HOOK1 were significantly associated with 16, 14, and 12 subtypes of immune cells, respectively. Fewer immune cell subtypes were associated with HIST1H4C and LOC730167, with only four and six subtypes. CARD17 and GBP5 exhibit a positive correlation with activated dendritic cells and a negative correlation with memory B cells, effector memory CD4 T cells, and central memory CD4 T cells. Combined with the expression of characteristic biomarkers and diagnostic efficiency, we suggest that immune cells may be regulated by GBP5 and CARD17 in EPTB progression.

### 3.8 Validation of GBP5 and CARD17 expression in clinical samples

To validate the expression of GBP5 and CARD17 in clinical samples, we conducted qPCR on samples from 10 EPTB patients and 10 healthy controls. The qPCR results demonstrated a significant upregulation of GBP5 (*p* = 0.029) and CARD17 (*p* < 0.001) in EPTB compared to the control group (Figures 6D, E). These findings are consistent with the expression data from the GEO dataset, further confirming that these genes may play a critical role in EPTB and can serve as a potential biomarker.

## 4 Discussion

Tuberculosis is the major infectious disease causing death worldwide. EPTB is an infectious disease caused by Mtb infection of organs other than the lungs (Sharma et al., 2021). Currently, studies on TB diagnosis mainly focus on PTB, and the diagnosis of EPTB from diseased tissue remains a challenge. Highly accurate blood tests are needed to improve the diagnosis of EPTB and to initiate anti-tuberculosis treatment promptly. Several studies have focused on EPTB diagnostic markers. Matrix metalloproteinases (MMPs) and tissue inhibitors of metalloproteinase (TIMPs) were found to be pathological regulators of TB, with MMP-13 and TIMP-2 differentiating EPTB from latent TB and healthy controls (Kathamuthu et al., 2020). Five inflammatory biomarkers (MIG, IP-10, MIF, CCL22 and CCL23) can be used to monitor the efficacy of EPTB treatment (Ambreen et al., 2021). Serum immunoglobulin response to cell wall products of Mycobacterium may be a valuable instrument for monitoring treatment response to PTB or EPTB in children and adolescents (Dos Santos et al., 2020). Although there have been advances in the diagnosis of EPTB, studies on immune-related serum biomarkers are still limited (Perumal et al., 2020; Liang et al., 2021). Thus, EPTB diagnostic biomarkers and their correlation with immune cell infiltration were investigated in this study.

Previous studies using the GSE83456 dataset have focused on building diagnostic models for PTB (Zhu and Liu, 2023) or active TB (Li et al., 2023). Its researches in EPTB are mainly about bone tuberculosis's diagnosis biomarkers (Liang et al., 2021) and molecular mechanism (Liang et al., 2022). In this study, 110 DEGs were screened using EPTB serum samples from GSE83456. Combined with WGCNA, the scope was further narrowed to 96 DEGs. GO analysis revealed that the 96 DEGs were mainly enriched in biological processes such as defense response and response to biotic stimulus. KEGG analysis suggests that EPTB-related pathways are mainly NOD-like receptor signaling pathway and various virus-related pathways closely related to immunity. Chronic viral infections increase the severity of Mtb co-infection (Xu et al., 2021). At present, studies on the mechanism of TB caused by Mtb infection have focused on immune escape (Zhai et al., 2019), cellular pyroptosis (Qu et al., 2020) and epigenetics (Batista et al., 2020). In this study, the enrichment analysis results suggest that the human immunity to EPTB may be more similar to viral infections than to common bacterial infections, but experimental validation is still needed.

LASSO regression and SVM-REF further identified five key DEGs (GBP5, HIST1H4C, CARD17, LOC730167 and HOOK1). The GSE144127 dataset was used for validation, including gene expression and diagnostic efficiency. The results revealed that GBP5 (AUC = 0.833) and CARD17 (AUC = 0.763) still had high diagnostic efficiency and may be critical biomarkers for EPTB. Guanylate binding protein 5 (GBP5) belongs to the IFN-γ inducible GTPase family and functions in host defense and inflammatory responses (Fujiwara et al., 2016). The GBP5 gene promotes NLRP3 inflammatory vesicles that exhibit a pronounced pathogen-host defense response (Shenoy et al., 2012). The GBP5 gene also regulates AIM2 inflammatory vesicle expression, triggering caspase-1-dependent pyrolysis and releasing the IL-1β and IL-18 (Meunier et al., 2015). Differential expression of GBP5 between TB and LTBI has been demonstrated by blood transcriptomics (Berry et al., 2010) and PCR (Laux da Costa et al., 2015). However, the association between GBP5 and EPTB is less well studied and needs further validation. Caspase recruitment domain 17 (CARD17) is closely associated with regulating protein hydrolase pro-1P maturation and release in inflammation (Lamkanfi et al., 2004). The latest study found that CARD17 was up-regulated in active tuberculosis by bioinformatic analysis, with a ROC curve showing 85% sensitivity, 90% specificity, and an AUC value of 0.96 (Natarajan et al., 2022). Recently, a blood RNA sequencing study on drug-resistant tuberculosis found that CARD17 was upregulated in multi-drug/rifampin resistant TB (Madamarandawala et al., 2023). Experimental studies on CARD17 are scarce, and there are no reports on the expression and mechanism of action of CARD17 in EPTB. We validated GBP5 and CARD17 expression in clinical samples by qPCR. The qPCR result showed that the expression of GBP5 and CARD17 was significantly up-regulated in EPTB patients compared to controls. This is consistent with the expression data in the GEO dataset. It is suggested that GBP5 and CARD17 could serve as potential biomarkers for EPTB and deserve further exploration.

The immune response to Mtb infection in different parts of the organism is diverse and complex (Consonni et al., 2022). As a consequence, safe and effective treatment requires precise control of the immune system. Using ssGSEA to assess immune cell infiltration and correlate it with the hub gene can contribute to diagnosing and treating EPTB. The infiltration of 17 subtypes of immune cells was significantly different compared to the control group. Five key genes, CARD17, GBP5, HOOK1, LOC730167 and HIST1H4C, were significantly associated with 16, 14, 12, 6, and 4 subtypes of immune cells, respectively. Memory B cell, Effector memory CD4 T cell, Central memory CD4 T cell and Activated dendritic cell were associated with more than two key genes (*p* < 0.001). HOOK1 is strongly associated with M2 macrophages in endometriosis (Wang et al., 2023), and GBP5 is associated with a high infiltration of B-cells, CD4+ T-cells, CD8+ T-cells, and NK-cells in small cell lung cancer (Tong et al., 2023). These high correlations suggest an important role for these genes in immune regulation and inflammation. Several studies of immune cells in EPTB have focused on CD4+ T cells. Mtb replication delays the initiation of CD4 T cells (Xu et al., 2021). Differential expression of Mtb-specific CD4+ T cell activation markers distinguishes EPTB from latent infection (Silveira-Mattos et al., 2020). CD4+ T cells can spread EPTB early, promote TB development, and maintain multi-effect functions of CD8+ T and CD3– lymphocytes (Yao et al., 2014). Increased memory CD4+ T cell response frequency in previously treated EPTB patients (Barreto-Duarte et al., 2020). Research on Memory B and dendritic cells is sparse and awaits our experimental additions. The evidence mentioned previously, and our current findings suggest that infiltrating immune cells are critical in EPTB and should be the focus of future research.

This study has limitations. While LASSO seeks sparse solutions and handles the observed data well, it is an ℓ1 penalized least squares regression method. To address the potential issue of multicollinearity, the Elastic Net (ENET) combines ℓ1 and ℓ2 penalty terms, which could be considered for use in subsequent studies (Zou and Hastie, 2005). Moreover, to mitigate overfitting associated with LASSO regression, we have introduced an SVM-RFE model that employs intersection taking to identify key genes. To further prevent overfitting, methods such as Smoothed Truncated Absolute Deviation (SCAD) and Adaptive Lasso (aLasso) could also be considered (Ejaz Ahmed, 2016; Ahmed et al., 2023). In addition, the GSE83456 control group is a healthy control. In contrast, the GSE144127 control group is a control for other diseases (including pneumonia and lung cancer), whose reproducibility needs to be further verified. Although we performed qPCR validation, the sample size was small and further prospective studies with larger sample sizes are needed to validate the findings.

## 5 Conclusions

In summary, we first obtained 96 EPTB characteristic genes based on WGCNA. Then, five key DEGs (GBP5, HIST1H4C, CARD17, LOC730167, and HOOK1) were screened by LASSO regression and SVM-REF. The AUC, area under the ROC curve, was selected as a performance metric. The results showed that GBP5 (AUC = 0.833) and CARD17 (AUC = 0.763) had high diagnostic efficiency. qPCR results confirmed that GBP5 and CARD17 were higher expressed in EPTB. They could be selected as diagnostic biomarkers for EPTB. The analysis of immune cell infiltration revealed that CARD17 and GBP5 are positively correlated with activated dendritic cells and negatively correlated with memory B cells, effector memory CD4 T cells, and central memory CD4 T cells. The immune cell dysregulation in EPTB is characterized by the downregulation of central memory CD4 T cells, memory B cells, and effector memory CD4 T cells, in contrast to the upregulation of activated dendritic cells. In the future, we will focus on exploring the diagnostic value and molecular mechanism of GBP5 and CARD17 in EPTB through cellular and animal experiments. We hope to provide new ideas and theoretical basis for the diagnosis and treatment of tuberculosis.

## Data availability statement

The original contributions presented in the study are included in the article/supplementary material, further inquiries can be directed to the corresponding author.

## Author contributions

YW: Conceptualization, Data curation, Funding acquisition, Methodology, Resources, Writing—original draft. FJ: Conceptualization, Writing—review & editing. WM: Formal analysis, Resources, Writing—review & editing. YY: Writing—review & editing. WX: Conceptualization, Data curation, Methodology, Project administration, Resources, Writing—review & editing.
